# Postoperative Weight Gain within Enhanced Recovery after Cardiac Surgery

**DOI:** 10.3390/jcdd10060263

**Published:** 2023-06-16

**Authors:** Alexandra Krüger, Anna Flo Forner, Jörg Ender, Aniruddha Janai, Youssef Roufail, Wolfgang Otto, Massimiliano Meineri, Waseem Z. A. Zakhary

**Affiliations:** 1 Heart Center Leipzig, University of Leipzig, Strümpellstraße 39, 04289 Leipzig, Germany; ak85qape@studserv.uni-leipzig.de; 2 Department of Anesthesiology and Intensive Care Medicine, Heart Center Leipzig, Strümpellstraße 39, 04289 Leipzig, Germany; anna.floforner@helios-gesundheit.de (A.F.F.); joerg.ender@helios-gesundheit.de (J.E.); aniruddha.janai@helios-gesundheit.de (A.J.); massimiliano.meineri@helios-gesundheit.de (M.M.); 3 Health Sciences, Faculty of Science, Waterloo Campus, Wilfrid Laurier University, Waterloo, ON N2L 3C5, Canada; rouf6200@mylaurier.ca; 4 Department of Cardiac Surgery, Heart Center Leipzig, Strümpellstraße 39, 04289 Leipzig, Germany; wolfgang.otto@helios-gesundheit.de

**Keywords:** ERACS enhanced recovery after cardiac surgery, weight gain, fluid overload, postoperative outcome

## Abstract

Optimal fluid therapy during perioperative care as part of enhanced recovery after cardiac surgery (ERACS) should improve the outcome. Our objective was finding out the effects of fluid overload on outcome and mortality within a well-established ERACS program. All consecutive patients undergoing cardiac surgery between January 2020 and December 2021 were enrolled. According to ROC curve analysis, a cut-off of ≥7 kg (group M, n = 1198) and <7 kg (group L, n = 1015) was defined. A moderate correlation was shown between weight gain and fluid balance r = 0.4, and a simple linear regression was significant *p* < 0.0001, R^2^ = 0.16. Propensity score matching showed that increased weight gain was associated with a longer hospital length of stay (LOS) (L 8 [3] d vs. M 9 [6] d, *p* < 0.0001), an increased number of patients who received pRBCs (L 311 (36%) vs. M 429 (50%), *p* < 0.0001), and a higher incidence of postoperative acute kidney injury (AKI) (L 84 (9.8%) vs. M 165 (19.2%), *p* < 0.0001). Weight gain can easily represent fluid overload. Fluid overload after cardiac surgery is common and is associated with prolonged hospital LOS and increases the incidence of AKI.

## 1. Introduction

Fluid overload after cardiac surgery (CABG) is common and increases postoperative complications [1]. To date, its impact within an Enhanced Recovery after Cardiac Surgery (ERACS) program has not been well studied. We can expect less fluid overload by applying ERACS elements such as liberal preoperative oral fluid intake, early extubation, early postoperative oral intake, and early mobilization.

Goal directed fluid therapy (GDT) has been recommended during ERACS [2] within the early postoperative period, between 8 h [3] and 24 h [4]; however, it has only shown weak impact on mortality through reduced morbidity and hospital length of stay [5]. Nevertheless, GDT was not consistently associated with a reduction in fluid intake [3].

Fluid overload could be minimized using a restrictive fluid therapy protocol. However, studies comparing liberal versus restrictive fluid therapy in cardiac [4] and non-cardiac surgeries [6,7] showed non-superiority in fluid-restricted groups.

Postoperative fluid overload can be quantified either by calculating the fluid balance or by measuring postoperative weight gain. The fluid balance calculation seems to be more precise but time consuming and less reliable due to charting errors and gaps [8]. Conversely, weight gain is more robust and easier to interpret [9].

The aim of this retrospective study was to assess the impact of fluid overload on outcome and mortality in a well-established ERACS program.

The primary endpoints are the incidence of weight gain after cardiac surgery and the correlation between postoperative weight gain and fluid balance. The secondary endpoints are the effects of fluid overload on hospital length of stay (LOS), fast track failure (FTF), acute and chronic kidney injury (AKI), postoperative pulmonary complications, packed red blood cells (pRBCs) transfusion, and mortality.

## 2. Materials and Methods

This retrospective observational study was performed at a university affiliated Heart Center and began after approval by the local ethics committee (registration number 552/20-ek).

We selected all consecutive patients who followed the Leipzig ERACS protocol from January 2020 to December 2021 undergoing elective or emergency cardiac surgery. Exclusion criteria were: patients ineligible for ERACS, younger than 18 years, non-cardiac surgery patients (e.g., PM, ICD, TAVR), and incomplete data.

**Figure 1:**
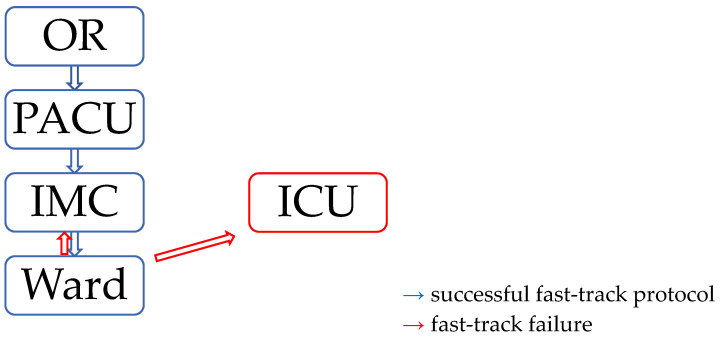
Fast-track pathway. OR = operating room, PACU = postanesthetic care unit, IMC = intermediate care unit, ICU = intensive care unit.

Patients without any complications were transferred to the postanesthetic care unit (PACU) directly from the operating room (OR). Once in the PACU, they were stabilized and extubated, hereafter transferred to the intermediate care unit (IMC) on the same day (POD 0). Patients were then transferred to the ward at the earliest on POD 1. The definition of a fast-track failure (FTF) is the unplanned transfer from the ward to the ICU or IMC at any time during the same hospital stay (Figure 1).

### 2.1. Pre-and Intraoperative Management

Patients were allowed to drink clear fluids, e.g., water, clear juices, and tea up to 2 h before surgery.

After anesthesia induction, a three-lumen central venous catheter and an 8.5 F introducer sheath were placed in the internal jugular vein under ultrasound guidance, and an arterial catheter was inserted for blood pressure monitoring. Body temperature was measured through a nasopharyngeal temperature probe and core temperature through a urinary catheter. Arterial and central venous blood gas analysis including hemoglobin (Hb), hematocrit (Hct), serum lactate, and central venous oxygen saturation (ScvO_2_) were collected every 30 min. A transesophageal echocardiography (TOE) probe was inserted in all patients, if not contraindicated (esophageal pathology). Swan-Ganz catheters were spared to patients with a low ejection fraction (EF) of ≤35% or impaired right ventricle (RV) function or tricuspid valve (TV) surgery. More details regarding anesthesia and postoperative analgesia were published previously [10,11].

The blood transfusion threshold was a hematocrit less than 20% during CBP and less than 25% after weaning from CBP and during off-pump surgery. Cell saver CATSmart^®^ (Fresenius Kabi AG, Bad Homburg, Germany) was used in all patients.

The following data were routinely collected intraoperatively: the amount of crystalloid/colloid infusion, packed red blood cells (pRBCs), fresh frozen plasma (FFP), platelets, cell saver, and fluid volume of the heart-lung machine during surgery (priming, cardioplegia). The outputs included urine output, remaining volume of the heart-lung machine, and ultrafiltration.

### 2.2. Perioperative Fluid Management

**Figure 2:**
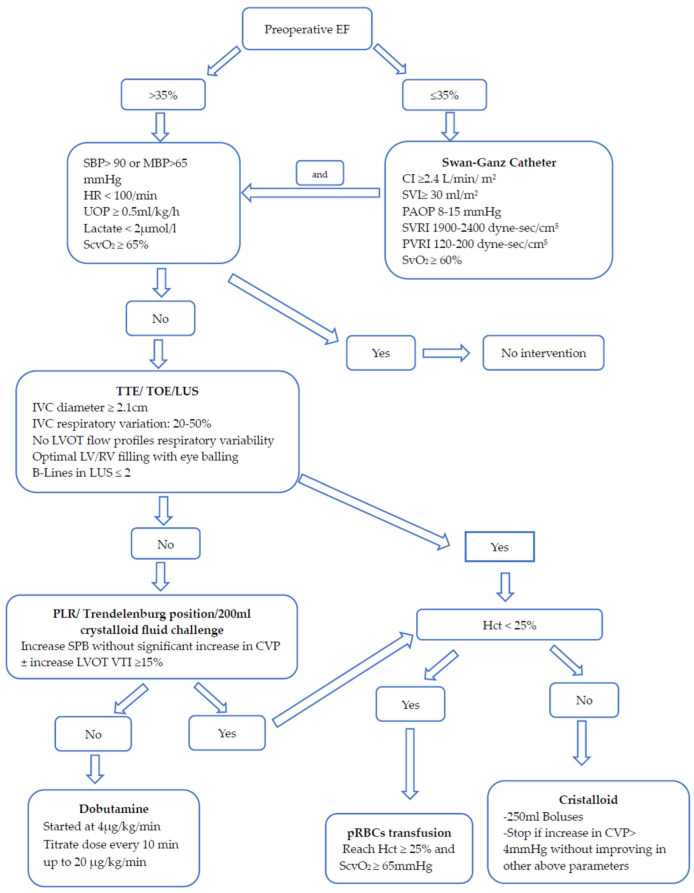
Fluid management algorithm. EF—ejection fraction; SBP—systolic blood pressure; MBP—mean blood pressure; UOP—urinary output; ScvO_2_—central venous oxygen saturation; SvO_2_—mixed venous oxygen saturation; IVC—inferior vena cava; PAOP—pulmonary artery occlusion pressure; SVRI—systemic vascular resistance index; PVRI—pulmonary vascular resistance index; CI—cardiac index; SVI—stroke volume index; TTE—transthoracic echocardiography; TOE—transesophageal echocardiography; LUS—lung ultrasound; PLR—passive leg raising; LVOT—left ventricular outflow tract.

Patients were treated with a specific fluid management algorithm starting intraoperatively and until transfer to the ward (Figure 2). The Swan-Ganz catheter (if indicated) was removed in the PACU if the patient had normal parameters; otherwise, the patient would have been transferred to ICU. Fluid therapy was monitored by the mean blood pressure (BP), heart rate (HR), urinary output (UOP), serum lactate, central venous oxygen saturation (ScvO_2_), TOE, transthoracic echocardiography (TTE), lung ultrasound (LUS), and hematocrit level (Hct). Swan-Ganz parameters were added for patients with reduced EF. The passive leg raising (PLR) maneuver, Trendelenburg position, or fluid challenge test using 200 mL of electrolyte crystalloids solution, e.g., Jonosteril^®^ (Fresenius Kabi, Bad Homburg, Germany), are used if the TTE, TOE, and LUS results are not conclusive (Figure 2).

### 2.3. Postanesthetic Care Unit (PACU) and Intermediate Care Unit (IMC) Management

Criteria for PACU eligibility were: hemodynamic stability (±low-dose inotropic support), no severe bleeding, core temperature ≥ 36 °C, and clinical judgement and communication between anesthesiologist and surgeon [12,13].

The PACU, managed by the department of anesthesia, includes 8 beds and is staffed with a physician to patient ratio of 1:4 and a nurse-to-patient ratio of 1:3. The PACU opening hours were from 10:00 a.m. to 10:30 p.m. only during weekdays.

Intraoperative fluid therapy monitoring continued in the PACU. Arterial and venous blood gas analysis and UOP were documented hourly. Combined transthoracic echocardiography (TTE) and lung ultrasound (LUS) imaging were performed for all patients at least twice in the PACU (before extubation and before transfer to the IMC) and if otherwise indicated (e.g., hemodynamic instability).

Patients were extubated after fulfilling the predefined extubation criteria [11], and thereafter, a noninvasive ventilation was performed for half an hour. Transfer to the Intermediate Care Unit (IMC) was usually done after monitoring the patient 2–3 h after extubation. The transfer criteria were: a fully awake and alert patient without neurologic deficit, hemodynamically stable with no or minimal inotropic support, acceptable blood gas analysis (PaO_2_ > 90 mmHg and PaCO_2_ < 46 mmHg, SpO_2_ > 96% on O_2_ flow 2–6 L/min), urinary output ≥ 0.5 mL/kg/h, no significant bleeding (<50 mL/h), normal serum lactate, normal SvO_2_, cardiac enzymes, and chest X-ray [12].

In the IMC, patients are fully monitored including invasive BP. Blood gas analysis was assessed every 3 h and POCUS (point of care ultrasound) if indicated.

Postoperative fluid intake (crystalloids/colloids, pRBCs, FFP, platelets, oral intake, medications) and output (blood loss, urine output (UOP), feces) were collected until discharge from the IMC to the ward, where the first postoperative weight was measured, which mostly represented the maximum recorded postoperative weight.

### 2.4. ERACS Protocol

We started with the fast-track protocol in November 2005 with 3 beds in the PACU completely separated from the ICU. The early experimental results were published in 2008 [13]. Many aspects of this concept were tested and published [10,11,12,14,15,16,17]. This protocol was dynamically developed to match ERACS evolution over the time. Fourteen from the 21 elements of perioperative ERACS bundles, guidelines by Engelman et al. [2] were already applied in our protocol (tranexamic acid; perioperative glycemic control; a care bundle to reduce surgical site infections; goal-directed therapy; a multimodal, opioid-sparing, pain management plan; avoid hypothermia after CBP; active maintenance of chest tube patency; post-operative systematic delirium screening; an ICU liberation bundle; early extubation strategies; chemical thromboprophylaxis; a clear liquid diet may be considered to be continued up until 4 hours before general anesthesia; routine stripping of chest tubes is not recommended; hyperthermia (>37.9 °C) should be avoided). Many of the preoperative elements are still not adequately applied. We were also applying some non-graded elements suggested by Zaouter et al. [18]. Yearly, we are adding more elements to the protocol. We used to use the Vigileo^®^ system (Edwards Lifesciences Corporation, Irvine, CA, USA) for all off-pump CABG for continuous stroke volume variation and cardiac index monitoring. Since 2018, intraoperative TOE, with certain indications for a Swan-Ganz catheter (see above), replaced Vigileo^®^.

### 2.5. Variables

The patients’ body weight was measured preoperatively as the baseline value and after transfer from the IMC to the ward.

Weight gain is defined as the difference between the preoperative weight and maximum postoperative weight.

Complex surgery is defined as any surgery including more than one procedure, e.g., CABG + Valve/s or 2 or more valves.

Postoperative respiratory complications included any cause of pneumonia, ARDS, and severe airway edema. The categorization of acute kidney injury follows the KDIGO stadium classification [19]. According to the KDIGO guidelines, chronic kidney disease was used to define persistence of kidney damage for more than 3 months.

Calculated fluid balance is defined as the mathematical subtraction of every single fluid output from every single fluid input. Measured fluid balance is the sum of all fluid balances documented in the nursing documentation sheet.

### 2.6. Statistical Analysis

Data were retrieved from the clinical information system iMedOne^®^ (Deutsche Telekom Healthcare and Security Solutions GmbH, Bonn, Germany) and the machine-readable patient’s chart Medlinq^®^ (Medlinq Softwaresysteme GmbH, Hamburg, Germany). Anesthesia and PACU observation sheets were corrected manually by the anesthesiologist directly after scanning. Data of cardiopulmonary bypass were documented by a special machine operator in the Connect Manager (LivaNova; London, UK). SPSS (SPSS^®^ Statistics 25.0; Chicago, IL, USA) and StatsDirect (StatsDirect^®^ version 3.3.5, StatsDirect Ltd., Cheshire, UK) were used for statistics.

Due to a number of studies with a difference in the time of collecting postoperative weight [1,8,9,20,21,22,23,24], ROC curve analysis for postoperative acute renal injury was used to determine the cut-off point of weight gain between both groups. Continuous variables were assessed for normal distribution using the Shapiro–Wilk test. The continuous data are expressed as mean ± SD and compared using a Student’s paired t-test in case of normal distribution or a Mann–Whitney U test for data not normally distributed. Categorical data were expressed as numbers (proportion) and compared using the X^2^ test or Fisher’s exact test where appropriate. Means of maximum weight gain for each postoperative day (POD) were compared with a one-way ANOVA with a Tukey–Kramer post-hoc test to show the differences between each pair. A *p*-value < 0.05 was considered statistically significant. A Pearson correlation coefficient (r) and simple linear regression was used for the relationship between weight gain and fluid balance.

According to our sample size analysis, 1538 patients had to be included if the hospital length of stay would have been shorter by one day (15 days vs. 14 days) ± 7 with a power of 80% and *p* = 0.05.

Propensity score matching was performed to minimize the bias that might result from differences in demographic, preoperative, and intraoperative data, especially for the complexity of the surgeries. The variables used for the logistic regression model were: age, gender, day of maximum recorded weight, EuroScore II, surgery complexity, x-clamp time, CPB time, and length of surgery. We used one to one matching, paring each subject to the closest propensity score subject from the other group. Based on the pre-matching range of baseline variable differences, the maximum caliper width for pair-matching was defined as 0.2 of the pooled logit score standard deviation.

## 3. Results

**Figure 3:**
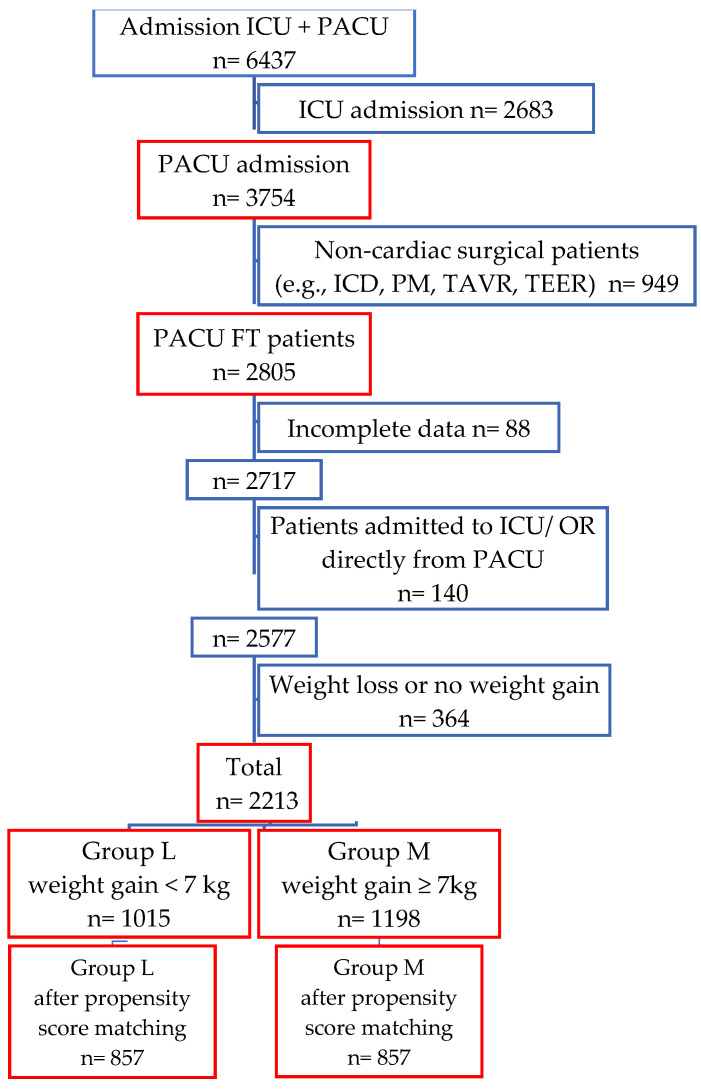
Patients’ flowchart.

A total of 6437 patients were operated between January 2020 to December 2021. In total, 3754 were admitted to the PACU. We included 2213 patients with complete data and who completed the ideal fast-track pathway from the PACU to the IMC and thereafter to the ward (Figure 3).

**Figure A1:**
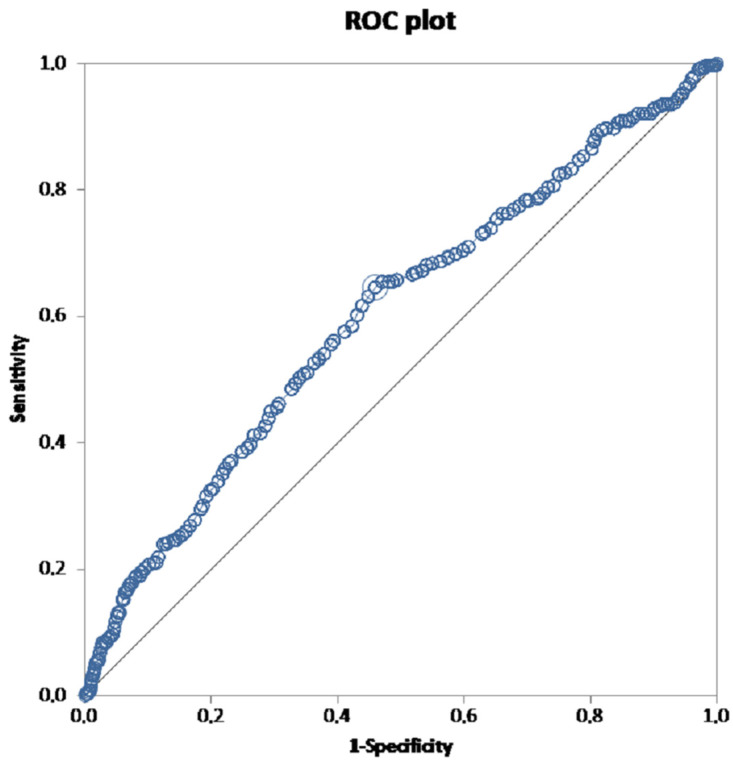
ROC plot for postoperative acute kidney injury.

According to ROC curve analysis (see Appendix A, Figure A1), a cut-off point of ≥7 kg (area under ROC curve = 0.601, PPV = 0.19, NPV = 0.88, sensitivity (95% CI) = 0.66, specificity (95% CI) = 0.48) was defined. Accordingly, patients were divided into 2 groups: group (L) with less weight gain (<7 kg, n = 1015 (46%)) and group (M) with more weight gain (≥7 kg, n = 1198 (54%)). After propensity score matching, each group included 857 patients.

Demographic data, baseline characteristics, and type of surgery before and after PSM are summarized in Table 1.

### 3.1. Weight Gain vs. Fluid Balance

The mean increase in weight was +7.6 ± 4.1 kg, while the mean intra- and postoperative fluid balance was +8.6 ± 3.7 L and the mean of the difference between fluid balance and weight gain was 1.0 ± 4.3 kg. Group L had a weight gain of 4.1 ± 1.8 kg, whereas group M had an average weight gain of 10.6 ± 3.1 kg. Only a moderate correlation was shown between weight gain and fluid balance (r = 0.4). A simple linear regression for weight change and fluid balance was significant (*p* < 0.001, R^2^ = 0.16). A total of 1675 patients was included in the fluid balance analysis (538 patients were excluded because of incomplete data). Inconsistency between the measured and calculated fluid balance was found in 26% of the patients.

### 3.2. Day of Maximum Collected Weight

**Figure 4:**
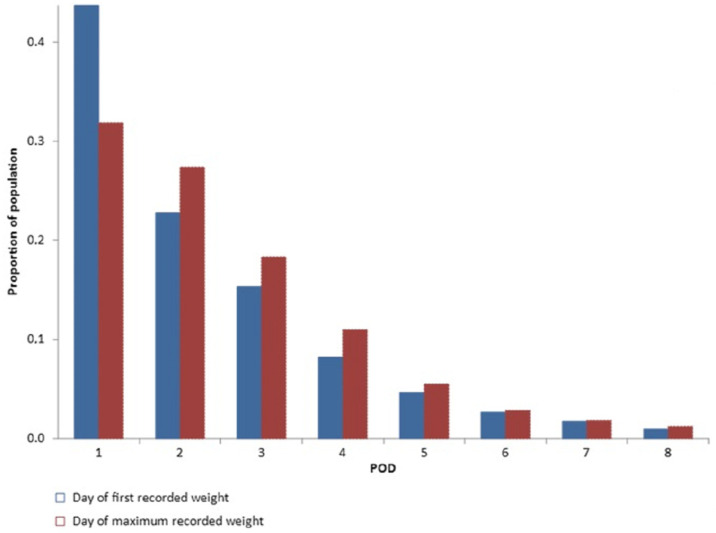
Day of first recorded weight vs. day of maximum recorded weight.

Figure 4 shows the proportion of patients for the first day of recorded weight and the day of maximum weight gain for each postoperative day (POD). Weight could be collected in 43% of the patients on POD1 and 23% on POD2. The maximum postoperative weight gain was concomitant to the first collected weight in 75% of patients weighed on POD1.

**Figure 5:**
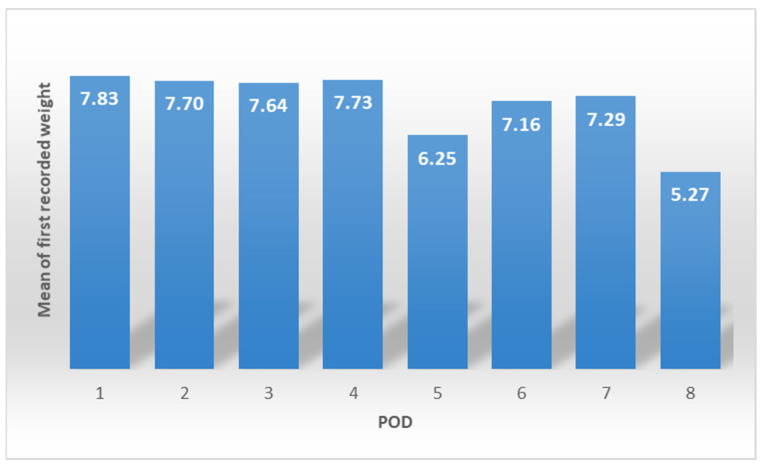
Mean of maximum weight gain.

The mean of maximum weight gain is comparable for patients weighed from POD1 to POD4 (7.6–7.8 kg) (Figure 5) with a drop in the mean on POD5 (*p* < 0.05 when compared with each day from POD1 to POD4).

**Figure 6:**
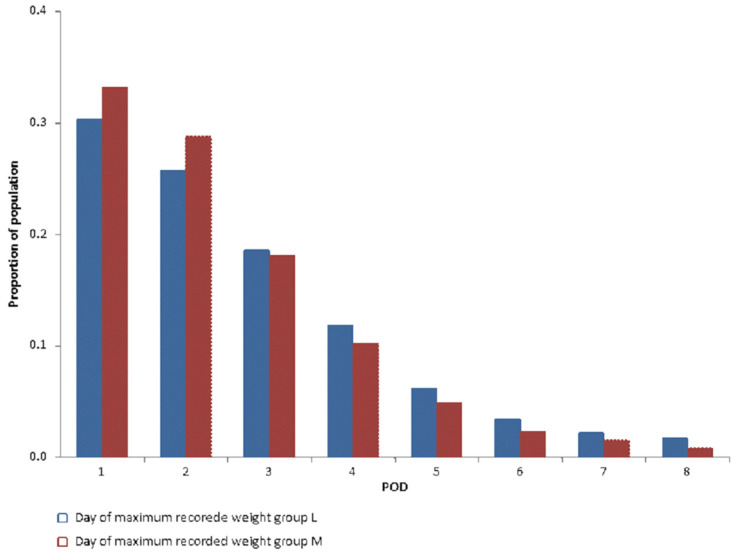
Comparison in day of maximum recorded weight between group L and M.

**Figure 7:**
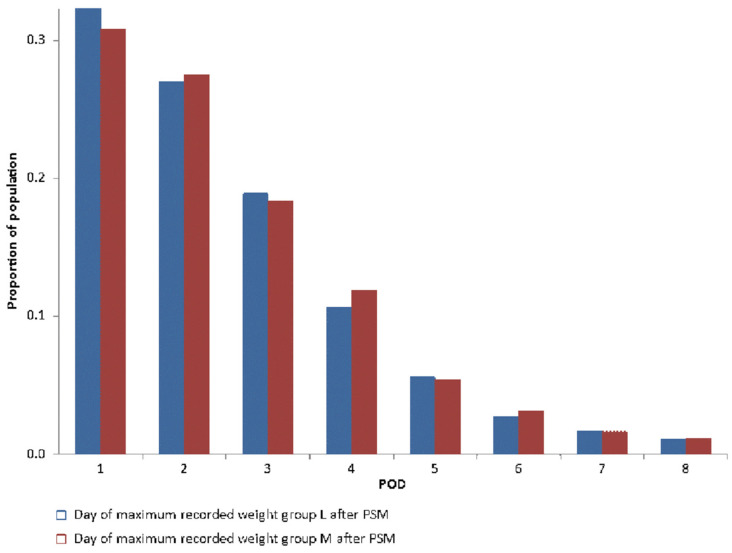
Comparison in day of maximum recorded weight between group L and M after PSM.

Figure 6 shows a significant difference (*p* < 0.001) in the day of maximum recorded weight. After PSM, no significant difference (*p* = 0.3) was found between groups (Figure 7).

Postoperative outcome and mortality are summarized in Table 2. Hospital length of stay was significantly longer in group M (L 8 [3] d vs. M 9 [6] d, *p* < 0.0001). Postoperative acute kidney injury at stages I and II, but not stage III, were significantly higher in group M. AKI was almost twice as high in group M compared to group L.

## 4. Discussion

In our study, 54% of our patients experienced postoperative weight gain ≥7 kg, which was associated with a longer hospital length of stay, increased number of received pRBCs, and higher incidence of postoperative acute kidney injury in enhanced recovery after cardiac surgery. We could find only a moderate correlation between the postoperative change of weight and perioperative fluid balance.

Engelman et al. [2] published the recommendations for ERACS in 2019. Many elements in the recommended bundles are potentially helpful to optimize the postoperative fluid status: perioperative glycemic control, GDT, early detection of kidney stress, early extubation, preoperative correction of nutritional deficiency, continued consumption of clear fluid until 2 h before surgery, and preoperative oral carbohydrates loading.

Unfortunately, there are few studies describing the effects of these elements on perioperative fluid management. GDT in cardiac surgery was described by Osawa et al. [3] and showed positive effects on postoperative major complications and hospital LOS but not on mortality. In comparison to our study, the fluid balance in Osawa et al. [3] was measured only until 8 h postoperatively, and GDT was unexpectedly associated with more fluid intake than the control group. Although a meta-analysis from Aya et al. [5] also showed a positive impact on complications and hospital LOS, it still could not reach a strong conclusion because of the limited data and heterogeneity of the methods used.

Liberal versus restrictive fluid therapy after surgery was also not well studied in cardiac surgery. Parke et al. [4] conducted a randomized controlled trial comparing the two groups after cardiac surgery. The overall fluid balance was lower in the intervention group (319 mL [−284–1274 mL] vs. 673 mL [38–1641 mL], *p* < 0.0001) that received less fluid boluses (1000 mL [250–2000] vs. 1500 mL [500–1500 mL], *p* < 0.0001); however, no impact was found on hospital LOS and postoperative complications. The hospital mortality rate was higher in the intervention group. Again, Parke et al. [4] limited their intervention to 24 h postoperatively, in contrast to our cohort who stayed in the IMC L 25 [46] h vs. M 24 [47] h in addition to a PACU stay L 235 [95] min vs. M 255 [85] min. Myles et al. [7] and Messina et al. [6] studied this issue in major abdominal surgery and found no impact between the two groups on major complications and postoperative mortality.

AKI stage I and II, but not III, were higher in our patients with more volume overload. Although it may be a causal bias (the patients were volume overloaded because of AKI, not vice versa), we do not think so because volume overload would be more obvious in AKI III, which is insignificantly different between both groups. Furthermore, preoperative serum creatinine was comparable between both groups. This is supported by Chen et al. [25] and Shen et al. [26] who found more AKI in volume overloaded and severely restrictive patients 48 h after cardiac surgery. In contrast, restrictive groups were associated with more renal complications than liberal fluid management in major abdominal surgery [6,7] but not in cardiac surgery [27]. A recent consensus report on adult cardiac surgery associated acute kidney injury (CSA-AKI) [28] mentioned both hypo- and hypervolemia as risk factors for CSA-AKI. They targeted euvolemia using thirteen strategies (pre-, intra- and postoperative) to prevent CSA-AKI. Nevertheless, AKI is an independent factor for prolonged hospital stay after cardiac surgery [29] and may explain the difference in hospital LOS between both groups.

No difference in the postoperative respiratory complications is also consistent with several studies [6,7,27]. The correlation between fluid overload and postoperative major complications was also demonstrated in CABG patients [1], which is in line with our results.

The difference in type of surgery between two groups is striking. There is significantly more aortic valve replacement (AVR) in group M. Patients with AV disease (mostly aortic stenosis) usually presented with LV hypertrophy and a degree of diastolic dysfunction. These patients, with good EF, tend to receive more fluids postoperatively as the sole treatment for any hemodynamic instability. POCUS examination of these patients mostly reveals LV hypertrophy and kissing papillary muscles that mandate fluid therapy. The presence of more on-pump CABG in group L is unexpected because the priming fluid, inflammation, and fluid shift effects of cardiopulmonary bypass may lead to fluid overload. Nevertheless, on-pump CABG is the most common surgical procedure in cardiac surgery, and the physicians may feel more confident in managing these patients, aiming for earlier hospital discharge compared to other types of procedures. Minimally invasive procedures including the thoracotomy approach and partial sternotomy were comparable in both groups. As in most minimally invasive cardiac surgery studies [30,31], these approaches are not inferior to the conventional approach regarding outcome. Olsthoorn et al. [31] showed a longer hospital LOS in a minimally invasive group, but we did not. Our patients were managed with the same ERACS protocol, regardless of type of procedure.

The discrepancy between the calculated and measured fluid balance and the correlation between fluid balance and body weight gain in our study is in concordance with the literature; Eastwood et al. [8] concluded the unreliability of fluid balance to reflect postoperative weight in cardiac surgery. Butti et al. [9] did not find a good correlation between fluid balance and weight gain after major abdominal surgery (r = 0.214, *p* = 0.19, vs. r = 0.4, *p* < 0.001 in our study). This could be explained by the complex calculation of fluid balance, which is more reliable for human error and is demonstrated in our study by the difference between the calculated and measured fluid balance of 26%. Furthermore, the insensible fluid loss is either ignored or imprecisely calculated. Therefore, we believe that weight gain is more representative of fluid balance after surgery. Köster et al. [20] showed the same results in ICU patients, preferring weight gain over fluid balance measurements.

A higher number of patients receiving transfusion can be noticed in our group M in contrast to group L. As per departmental SOPs, we have a restrictive threshold for transfusion. However, 36% of the group L still received at least one pRBC, which is still in the range of pRBCs transfused in major randomized studies comparing restrictive vs. liberal transfusion after cardiac surgery (52.3% and 25%) [32,33].

Physician compliance and adherence to fluid management protocols could not be assessed because of the nature of retrospective studies. Implementation of fluid management protocols (and all other ERACS elements) are reinforced with regular audits, availability of diagnostic tools (ultrasound machines), and documentation in the local manual for cardiac anesthesiologists. Nevertheless, “lack of compliance” with failure to adhere to the standardized protocol could not be excluded as reason for fluid overload. In the anesthesia setting, implementation of quality indicators was confronted with poor employee compliance [34].

The main limitation of our study is the retrospective design through which we cannot fully exclude the risk of unknown biases. This study did not take into account intraoperative insensible fluid loss and intraoperative bleeding not processed through the cell saver system. We could not precisely assess adherence to the fluid management protocol. It is difficult to compare hospital LOS in this study with studies from other countries. In Germany, the hospital LOS depends not only on the patients’ general condition, but also on the German payment system. Minimum hospital LOS between 4–12 days, according to the cardiac procedure, is required for full payment from health insurance companies. In 2018, the average length of stay for cardiac surgery patients in Germany was 11 days [35,36].

## 5. Conclusions

Volume overload after cardiac surgery is also quite common within a well-established ERACS program, and it is associated with a prolonged hospital length of stay, higher incidence of postoperative AKI, and blood transfusion. Weight gain is likely to estimate the fluid overload in a better way compared to complex fluid balance calculations. Individual ERACS elements must be prospectively studied to examine its effect on postoperative fluid overload.

## Figures and Tables

**Table 1:** Baseline characteristics and demographic data; values in mean ± SD or median [IQR] or number (%); {propensity score matching (PSM) results}.

	L n = 1015 {after PSM n = 857}	M n = 1198 {after PSM n = 857}	*p* Value
Age (y)	62.7 ± 11.6 {63 ± 11.5}	64.3 ± 11.8 {64 ± 11.6}	0.02 {0.129}
Female gender	238 (23) {207 (24)}	331 (27) {234 (27)}	0.02 {0.15}
NYHA III and IV	353 (34) {311 (36)}	453 (37) {315 (37)}	0.1 {0.92}
Preoperative weight (kg)	85 ± 17 {85.3 ± 16.9}	84 ± 17 {84.3 ± 17.1}	0.137 {0.255}
BMI (kg/m^2^)	28.2 ± 4.7 {28 ± 4.7}	28 ± 5 {28.1 ± 5.1}	0.249 {0.486}
EuroSCORE II	1.82 ± 2.0 {1.6 ± 2.1}	2.0 ± 2.1 {1.6 ± 2}	0.547 {0.949}
Complex surgeries (CABG + Valve/s or 2 or more x Valve)	149 (14.6) {143 (16.6)}	283 (23.6) {153 (17.8)}	<0.0001 {0.565}
On-pump CABG	271 (26.6) {226 (26)}	221 (18.4) {165 (19.2)}	<0.0001 {0.0006}
OPCAB/MIDCAB	237 (23.3) {167 (19.5)}	155 (13) {149 (17.3)}	<0.0001 {0.289}
Isolated AVR	170 (16.7) {150 (17.5)}	314 (26) {210 (24.5)}	<0.0001 {0.005}
Isolated MVR	106 (10.4) {100 (11.6)}	147 (12) {98 (11.4)}	0.20 {0.939}
Minimal invasive approaches (Thoracotomy or partial sternotomy)	255 (25) {237 (27.5)}	317 (26.3) {227 (26.4)}	0.504 {0.624}
Thoracotomy approach	170 (16.7) {156 (18.2)}	169 (14) {129 (15)}	0.096 {0.091}
Partial sternotomy (superior)	85 (8.3) {81 (9.4)}	148 (12.3) {98 (11.4)}	0.003 {0.20}
Emergency surgery	127 (12.6) {101 (12)}	153 (12.9) {106 (12.5)}	0.905 {0.76}
Re-do operations	60 (6.2) {53 (6)}	83 (7.2) {56 (6.5)}	0.375 {0.781}
Preoperative ejection fraction (%)	56 ± 10.5 {56 ± 10.5}	57 ± 10.4 {57 ± 10.5}	0.082 {0.148}
Hypertension	761 (78.3) {643 (77.5)}	852 (75) {625 (73)}	0.47 {0.349}
DM	244 (24) {205 (24)}	276 (23) {196 (23)}	0.614 {0.648}
Preoperative hematocrit (%)	40.8 ± 5.4 {38.9 ± 4.9}	40.1 ± 5.8 {38.3 ± 4.9}	0.161 {0.001}
Preoperative hemoglobin (g/dl)	12.5 ± 2 {12.5 ± 1.6}	12.2 ± 2 {12.3 ± 1.6}	0.09 {0.001}
Minimal intraoperative hemoglobin (g/dl)	10.7 ± 4.8 {10.7 ± 5}	10.1 ± 3.2 {10.1 ± 2}	<0.0001 {<0.001}
Preoperative creatinine (mg/dl)	0.96 ± 0.02 {0.97 ± 0.69}	0.91 ± 0.01 {0.92 ± 0.3}	0.642 {0.02}
CPB duration (min)	71 ± 56 {76 ± 51}	87 ± 51 {78 ± 49}	<0.0001 {0.322}
x-clamp time (min)	40 ± 41 {46 ± 41}	55 ± 40 {47 ± 40}	<0.0001 {0.574}
Length of surgery (min)	183 ± 61 {184 ± 60}	192 ± 55 {188 ± 53}	0.002 {0.06}
Postoperative ventilation time (min)	106 ± 54 {106 ± 55}	115 ± 60 {115 ± 60}	0.015 {0.004}
PACU LOS (min)	240 [90] {235 [95]}	245 [90] {255 [85]}	0.006 {<0.001}
IMC LOS (h)	27.5 [47] {25 [46]}	23.1 [45] {24 [47]}	0.008 {0.625}
Weight gain (kg)	4.1 ± 1.8 {4.2 ± 1.8}	10.6 ± 3.1 {10.4 ± 3}	<0.0001 {<0.0001}

LOS—length of stay; CABG—coronary artery bypass graft; OPCAB—off-pump coronary artery bypass; MIDCAB—minimal invasive coronary artery bypass; AVR—aortic valve replacement; MVR—mitral valve replacement; DM—diabetes mellitus; CPB—cardiopulmonary bypass.

**Table 2:** Postoperative outcome and mortality after propensity score matching. Values in mean ± SD or median [IQR] or number (%).

	L n = 857	M n = 857	*p* Value	95% CI	RR
Hospital LOS	8 [3]	9 [6]	<0.0001	0.58 to 0.64	
In-hospital mortality	1 (0.1)	5 (0.5)	0.12		0.19
FTF	81 (9.5)	103 (12)	0.103		0.86
Postoperative acute kidney injury (AKI) total	84 (9.8)	165 (19.2)	<0.0001		0.63
AKI stage I	62 (8.4)	136 (15.8)	<0.0001		0.66
AKI stage II	9 (1)	24 (2.8)	0.013		0.54
AKI stage III	3 (0.3)	5 (0.6)	0.506		0.75
Postop chronic renal impairment	91 (11)	117 (14.5)	0.06		0.86
Any postoperative pulmonary complications	17 (2)	16 (1.8)	0.999		1.0
Number of patients who received 1 or more pRBCs	311 (36)	429 (50)	<0.0001		0.75

RR—relative risk; LOS—length of stay; FTF—fast-track failure; pRBCs—packed red blood cells.

## Data Availability

The datasets generated and analyzed during the current study are available from the corresponding author on reasonable request.
